# The role of the interaction network in the emergence of diversity of behavior

**DOI:** 10.1371/journal.pone.0172073

**Published:** 2017-02-24

**Authors:** Alan Godoy, Pedro Tabacof, Fernando J. Von Zuben

**Affiliations:** 1 Laboratory of Bioinformatics and Bioinspired Computing, School of Electrical and Computer Engineering, University of Campinas, Campinas, São Paulo, Brazil; 2 CPqD Foundation, Campinas, São Paulo, Brazil; 3 RECOD Lab, School of Electrical and Computer Engineering, University of Campinas, Campinas, São Paulo, Brazil; Consejo Nacional de Investigaciones Cientificas y Tecnicas, ARGENTINA

## Abstract

How can systems in which individuals’ inner workings are very similar to each other, as neural networks or ant colonies, produce so many qualitatively different behaviors, giving rise to roles and specialization? In this work, we bring new perspectives to this question by focusing on the underlying network that defines how individuals in these systems interact. We applied a genetic algorithm to optimize rules and connections of cellular automata in order to solve the density classification task, a classical problem used to study emergent behaviors in decentralized computational systems. The networks used were all generated by the introduction of shortcuts in an originally regular topology, following the small-world model. Even though all cells follow the exact same rules, we observed the existence of different classes of cells’ behaviors in the best cellular automata found—most cells were responsible for memory and others for integration of information. Through the analysis of structural measures and patterns of connections (motifs) in successful cellular automata, we observed that the distribution of shortcuts between distant regions and the speed in which a cell can gather information from different parts of the system seem to be the main factors for the specialization we observed, demonstrating how heterogeneity in a network can create heterogeneity of behavior.

## Introduction

“All animals are equal, but some animals are more equal than others.”

In the book Animal Farm [1], George Orwell tells us a story in which animals in a farm, tired of being exploited by humans, got rid of the farmer and created an egalitarian society, totally controlled by animals. Some time after such rebellion, however, a special class appeared—the pigs –, which did not produce any food and only took care of bureaucratic work. At one point the pigs acquired so many privileges and got so similar to humans that they replaced all revolutionary principles by just the one that opens this paper.

Apart from the political allegory expressed in the book, Animal Farm can draw our attention to a question relevant to many disciplines: how can a system composed of equal (or very similar) agents display qualitatively different behaviors? This is a particularly relevant question because, in most cases, instead of being only deleterious, as in Orwell’s book, differences in individual behavior may have positive effects. Consider, for instance, the human brain: while there are only a few hundred different types of neurons [2], the number of behavioral patterns in the brain is countless. Some neurons process auditory information, others are related to vision and others to empathy [2, 3]. Some people even have neurons that react only to images of specific celebrities, like Jennifer Aniston or Bill Clinton [4]. Human groups are another example of systems that can find some benefits in heterogeneity, as there is compelling evidence that diversity can improve a team’s performance in collective problem solving [5]. Indeed, human societies are remarkable for collectively solving complex problems, as the use of markets to organize labor division and control shared resources, and for learning and diffusion of information through science and culture [6, 7].

Heterogeneity is particularly intriguing in self-organized systems, as, having no central element controlling the role to be performed by each agent or how individual results should be combined, any necessary decision about task allocation or specialization is distributed among all agents [8].

Surely, in many cases heterogeneity is influenced by diversity in intrinsic features of its components—e.g.: the different types of neurons or genetic differences between humans in our two previous examples –, but in many situations these inner characteristics are not sufficient to explain the wide range of behaviors and specialization observed. A neuron is activated when one sees a picture of a celebrity not because there is a specific type of neuron that is good at identifying whether a person is famous or not, but as a consequence of its position within the neural network that connects the eye to the visual cortex to other regions of the brain [2]. Similarly, one would hardly agree, for instance, with the hypothesis of the existence of a “butcher gene” or a “lawyer gene”, i.e., that our genetic codes are the only factor determining the professional roles humans take in society. Physical and social environments have a crucial part in defining how we humans act and differentiate from each other [9, 10], so that the structure of our social connections has great influence on individuality [11].

Communication between agents is essential in self-organized systems [12] and it can happen according to a more or less rigid structure: in animal groups there are few restrictions limiting the potential contacts of an individual, so that an ant can interact with any other nestmate when looking for food sources or for potential new nests [13, 14]. In neural networks, on the other hand, synaptic connections are well delimited and stable in time [2, 15, 16], so that neurons can only transmit signals to their peers to which it is connected by an axon terminal. These synapses are persistent but dynamic, as connections can be created, tuned, or pruned to encode new knowledge or ability learned by the network.

**Fig 1:**
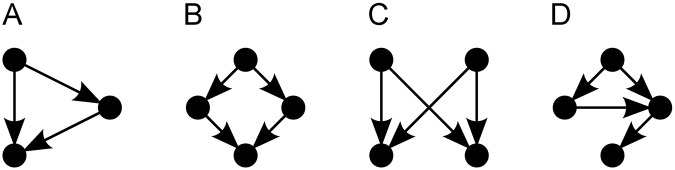
Motifs found in the neural network of the worm *C. elegans*. Motifs names: **(A)** Feed-forward loop, **(B)** Bi-fan and **(C)** Bi-parallel; motif **(D)** has no common alias. After Milo *et al.* [23].

Different patterns of connection can provide different functionalities in systems structured according to a network [17], like resilience to random failure of nodes (e.g.: neurons, people, computers) or connections (e.g.: synapses, social contacts, wires), more information storage capacity, ease of navigation within the network, influence concentration, and/or low average distance between each pair of nodes [18–21]. Local structures can have large effects on a network’s behavior: *motifs* (see Fig 1)—small patterns of connection that occur in a network more commonly than expected in a random network with similar characteristics—are believed to act as basic functional units in complex systems, having a similar role to that performed by logic gates in electronic circuits [22, 23].

Therefore, if we want to understand collective computation we must first understand how connections affect the flow and processing of information in complex systems. In this study, we want to shed some light on how network randomness can promote individual behavior heterogeneity in a system in which all individuals follow exactly the same rules, studying also how this randomness affects collective computation in complex systems and can induce specialization. For this, we combined a simple model of complex system known as *elementary cellular automaton* (ECA) with small-world networks—a family of networks capable of displaying some non-trivial characteristics exhibited by networked systems in the real-world –, using evolutionary computation to explore how individual behaviors and collective performance are correlated to communication structure, specially to the presence of direct connections between otherwise distant agents.

As we explain in the next section, most previous works studied computation in complex systems by analyzing global properties of cellular automata, which were obtained by the evolution only of rules followed by individuals. We, in turn, choose to evolve both networks and rules and to focus our analysis on the differences between behaviors of individual cells in a cellular automaton. For this we used information theoretical measures to describe such behaviors, and investigated how topological characteristics and local patterns of connections between individuals shape these differences.

## Background

As previously stated, we used in this work cellular automaton, a classic agent-based model. However, instead of following the common regular network, the communication in this study was structured according to a small-world network. If you are familiar with cellular automata, complex networks and previous works on the evolution of rules and networks for elementary cellular automata, you can gladly jump to the next section.

### Simple models for distributed systems

In this work, we used computation as an analogy to understand the information processing happening in self-organized systems. Currently, the most common computer architecture is the von Neumann architecture, in which one processor (CPU) has access to a memory (RAM) where program instructions and data are stored. Roughly speaking, the CPU fetches instructions from the memory and executes them, using this same memory to store all inputs and outputs of these computations. Complex and other massively distributed systems, however, are very different from such usual model of computation: while in a von Neumann architecture the CPU has access to the whole memory, in a complex system each individual (or processing unit) has complete access only to its own state (we can refer to this state as the individual memory) and can gather information solely from those individuals to which it is directly connected [8]. To exemplify such limitation, we can think about neurons: when deciding whether to fire or not, a neuron does not know the state of the whole brain but only of the neurons to which it is connected [2].

So, if we hope to study processes happening in complex systems we will need to use a model other than the von Neumann architecture. One of the simplest yet most powerful models for this kind of systems is the *cellular automaton* (known as CA). A CA is composed of a set of cells, each of which works like this:

- The cell has an *internal state*, which works as a (very) small memory—the number of possible states in which a cell may be is finite and fixed;
- In addition to its own state, the cell knows the current state of a given set of other cells, known as *neighbors* or contacts;
- At each step, the cell will update its state following a *rule table* that has as input the current state of the cell and of its neighbors and yields as output the next state for this cell.

Each cell may have a particular set of possible states or follow a different rule table [24]. However, the most common model, that was also used in this work, is the *uniform* cellular automaton, in which all cells are equal to each other. As each piece of this kind of cellular automaton is too simple, you might wonder about its ability to perform complex computation. Surprisingly (at least for some people), even for cellular automata in which the cells have only two possible states, there are combinations of neighborhoods and rule tables that can enable such CAs to solve each and every problem that can be solved by the most powerful computational model, the universal Turing machine [8, 25].

**Fig 2:**
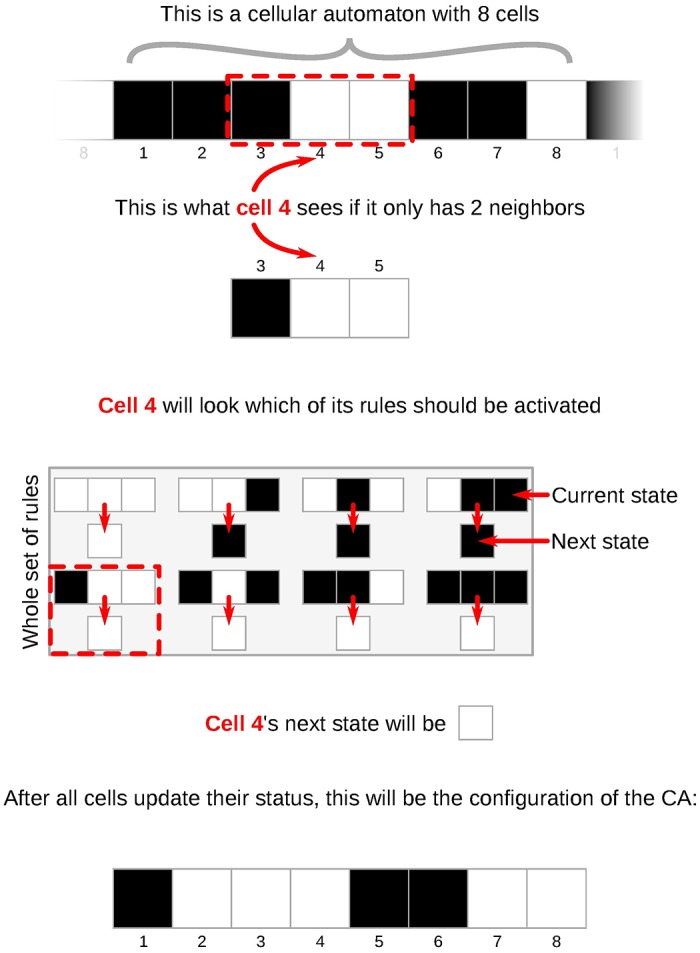
How elementary cellular automata work. Notice that all cells follow the same set of rules. The state of each cell is black (0) or white (1). The cellular automaton is uniform in the sense that all cells follow the same rule table to update their states.

**Fig 3:**
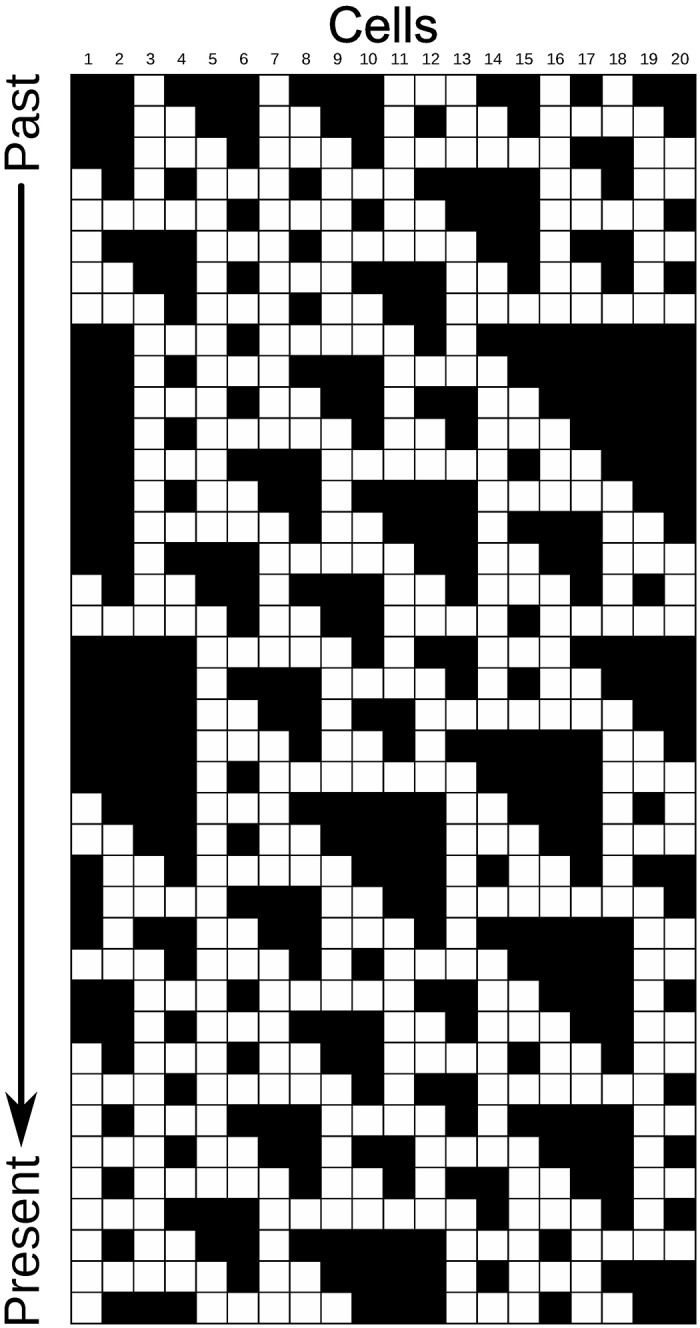
Space-time diagram of an ECA composed of 20 cells, each following the rule 110. See subsection “Searching good CAs” for an explanation on rule numbering. The initial state is exhibited at the top row. Cells 1 and 20 are neighbors.

The simplest “flavor” of cellular automaton is the elementary cellular automaton [26]. As shown in Fig 2, an ECA is composed of a fixed number of uniform cells, organized one beside the other, forming a (one dimensional) ring. Each cell can be in one of two states (0 or 1) and has direct contact only to their *k* closest peers, updating their states synchronously. Even though agents operate asynchronously in nature, this kind of CA has a dynamic rich enough to model many systems in the real-world. Even when *k* = 3 (i.e., a cell can only communicate to itself and its immediate neighbors to the left and to the right) some ECAs are able to perform universal computation [27], being some of the simplest machines able to do so. In Fig 3, you can see an example of the execution of one of such rules and the elaborate patterns it is able to produce. This combination of simplicity and ability to produce complex behavior, embedded within an inherently distributed system, is so impressive that some researchers, like Stephen Wolfram, see ECAs as key to explain how complexity arises from simple rules in nature [26]. With such model we can simulate idealized versions of complex systems to study the origins of *self-organized* processes and *emergent behaviors*—hard to predict behaviors that arise from the interaction of many individuals following simple rule –, being able, thus, to analyze the collective computation we see in systems like human brains and animal societies.

A model problem commonly used to study computation in cellular automata is the *density classification task* (also known by its acronym, DCT, or by a different name, *majority problem*) [8]: we initialize each cell in a CA with an arbitrary state, 0 or 1, and, after a fixed number of steps, all the cells must have converged to 1 if more than half of them were initialized with 1 (i.e., the initial density *ρ*_0_ was greater than 0.5), or 0 otherwise (*ρ*_0_ < 0.5). This sounds as a pretty simple problem, but it has been proven that no ECA with finite *k* can solve the DCT perfectly [28]. This is an easy task for a machine with access to the whole memory, but not for a distributed system where each piece knows only local information: you can understand the difficulty of such problem, for instance, by thinking about the challenge of knowing the average opinion of a country’s population by talking only to people who live near you.

Instead of reducing the interest for the DCT, the non-existence of a perfect solution for this task puts it in a privileged position for the study of complex systems, as it is both a very simple problem to formulate and a computationally difficult task. Thus, many researchers have looked for ECAs able to correctly perform density classification for as many initial configurations as possible, the first of which was Norman Packard. Packard analyzed DCT’s rule space [29] and used evolutionary computation to list some rules able to achieve good performance in that problem, studying their common characteristics [30].

The use of evolutionary techniques to search for efficient rules for the DCT (and other problems) was further explored by Melanie Mitchell, Peter Hraber, Rajarshi Das and James Crutchfield in a series of influential papers [31–33]. Two years after that, David Andre, Forrest Bennet and John Koza [34], using genetic programming, discovered the first computer-designed rule able to beat the performance of hand-coded ECAs. Before that, one of the best results was achieved by the rule GKL [35], which can be summarized as follows: if a cell is in state 0, then its next state is defined by the majority among itself, its nearest neighbor to the left and the neighbor three places to the left; if it is in state 1, symmetrically, it follows the majority among itself, its nearest neighbor to the right and the neighbor three places to the right.

In 1998, Juille and Pollack [36] obtained a better rule using a coevolutionary approach. Currently, due to an emergent phenomenon, two rules share the best results in the DCT problem [37]. While the best solution in grids with an even number of cells is still the JP rule, for odd-sized CAs the best results are obtained by the WO rule [38], which was found in 2008, combining a multi-level selection evolutionary algorithm with a bias favoring the selection of symmetric rules.

The results achieved by these rules are summarized in Table 1.

**Table 1:** Some of the rules with best performance in the DCT (in a grid with *N* = 149 and *k* = 7). Results are ordered according to the year they were discovered.

Rule	Year	Performance
GKL [35]	1978	81.6%
Mitchell et al. [31]	1993	76.9%
ABK [34]	1996	82.3%
JP [36]	1998	85.9%
WO [38]	2008	89.0%

### Networks in a nutshell

Real-world networks rarely are as organized as the ring topologies used in the studies we have just presented. The reality is much “messier”! So let us make a brief pause in our discussion about cellular automata and focus for a moment on networks.

**Fig 4:**
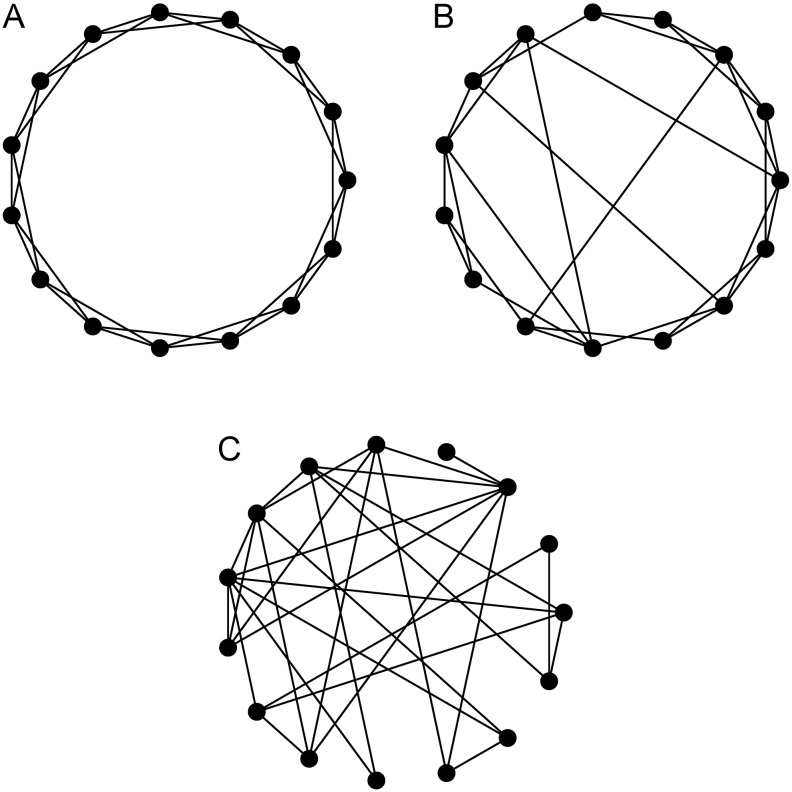
Graphs generated using the small-world model with different rewiring probabilities *p*. **(A)** *p* = 0.00, **(B)** *p* = 0.15 and **(C)** *p* = 1.00.

Until few years ago, networks seen in real systems, like neural and social networks, were considered too complex and, thus, were modeled as if they were completely random [19]. As we had access to more data, more computing power and better analytical tools, however, researchers started noticing that many real-world networks share some common properties that are not expected in purely random or regular networks. An example is the *small-world effect*, formally defined by Duncan Watts and Steven Strogatz [39] in a seminal paper published in 1998, which is the simultaneous occurrence in a network of a *small average distance* between each pair of nodes and a *high clustering coefficient* (i.e., a high probability that two vertices that are both neighbors to the same third vertex are also directly connected to each other). Watts and Strogatz showed that, while this effect is present, for instance, in human social networks, in power grids and in the neural network of the worm *Caenorhabditis elegans*, no lattice, ring or completely random graph is able to display both small average distances and high clustering. To produce more realistic networks they proposed a new model to generate networks, the *small-world model*, summarized in two components (see Fig 4):

- **Initialization:** the network is started as a ring in which each node is connected to its *k* closest neighbors;
- **Rewiring:** every edge in the initial network has a probability *p* of being *rewired*, i.e., of being disconnected from one of its ends and attached to a randomly chosen vertex (avoiding any self-loop or repeated edges).

By choosing a small value for *p* (usually less than 0.1), it is possible to preserve the local structure with high clustering of the initial ring, while the few long-distance *shortcuts* added are able to dramatically reduce the average distance between nodes.

The small-world effect is not the only common phenomenon missed by random and regular networks: the distribution of degrees (i.e., the distribution of the number of edges connected to each node) in many real-world networks deviate significantly from the Poisson or delta distributions expected for completely random graphs, lattices, rings and small-world networks. Instead, their degree distributions follow power laws—in these networks the probability that a random node has exactly *k* connections is given by *p*(*k*) ∝ *k*^−*γ*^, for a given positive *γ*. This result was published in 1999 by Albert-László Barabási and Réka Albert [40], after studying networks as those formed by citations between scientific publications, links between web pages and electric power grids. Like Watts and Strogatz, they also proposed a new network model, the scale-free model, which is able to produce networks with degrees following a power-law. Its basic components are the continuous inclusion of new nodes and edges and preferential attachment, a mechanism that selects nodes to receive new edges with probability proportional to the number of connections these nodes already have.

As we stated before, topologies with different characteristics can provide different functionalities to a system. For instance, the connectivity of networks with degrees following a power law has higher resilience against random removal of nodes, when compared to purely random graphs. However, this connectivity degenerates very quickly when the most connected nodes are removed, which makes them vulnerable to targeted attacks [19]. Initial studies also suggest that topologies combining scale-free networks and the small-world effect may be the result of an optimization process [41, 42], as they provide a good compromise between high connectivity between nodes and low demand for links. It is reasonable, thus, to suppose that the behavior of a distributed system, as an ECA, may differ a lot when we replace the usual regular topologies by more realistic networks to define how its units interact.

### The impact of topology on computation

Most of the research about collective computation has been centered on understanding the impact of individual behaviors on the global dynamics. Some works, however, studied the impact of modifying the topology connecting the individuals. Maybe the earliest work on this subject was the study of random Boolean networks (RBNs) by Stuart Kauffman [43]. In Kauffman’s original model, which was created to model genetic regulatory networks, each individual has a randomly defined Boolean activation function, being connected to each other according to a completely random topology. In these networks, we observe cyclic behaviors that may be classified as robust or chaotic, according to the system’s reaction to perturbations (which, in turn, is determined by the interplay between topological and behavioral parameters) [44].

More recently, in 1997, Moshe Sipper and Eytan Ruppin [45] studied a class of cellular automata that, differently from ECAs, allow each cell to evolve a different rule table. After evaluating how different topologies affect the ability of CAs to solve the DCT problem, they identified that a cellular automaton’s performance is strongly dependent on the average distance between its cells—the smaller the distance, the higher is the fitness. This is not an unexpected result: going back to our analogy of the DCT problem with opinion averaging, the insertion of long-range connections is similar to allowing you to talk not only to people in your neighborhood, but also to friends that live in different cities. The more shortcuts one has access to, the more one has access to diverse, less correlated opinions.

When studying small-world networks and their possible impacts on dynamical systems, Watts and Strogatz used this class of networks to define the neighborhood in ECAs, while keeping the individual behaviors fixed [39, 46]. They analyzed the effect of these topologies on the ability of such systems to solve the density classification task. They found that, for the same number of cells and average degree, such small-world ECAs were able to outperform even the best rules at that time which made use of the traditional ring topology.

Digging deeper on the effects of different neighborhood structures on the ability of cellular automata to solve computational problems, Marco Tomassini, Christian Darabos and Mario Giacobini [47, 48] used evolutionary computation to look for classes of topologies with which ECAs were able to achieve good performance in the density classification and synchronization tasks. As in Watts’ work, they kept fixed the rule used by the cells to update their states. The authors found that the networks able to produce the best results had some characteristics similar to small-world networks, both when they started the evolution from lattices or from random graphs. In agreement to Watts [46], the authors suggested that it is easier to get good results evolving networks when compared to evolving rules, as they obtained many ECAs able to solve more than 80% of the initial configurations, while it is very rare for lattices to achieve such performance. For instance, Crutchfield et al. [49] evolved high-scoring strategies only in 9 out of 300 runs of the evolutionary algorithm. A second interesting result they observed was that ECAs using small-world neighborhoods are more robust to random failures in cells (i.e., a cell updates its state to the incorrect output with probability *p*_*fail*_) than regular lattices. In subsequent works [50–52], they compared the effects of using small-world and scale-free networks to structure the communication in cellular automata and random Boolean networks, observing that, when solving the DCT or the synchronization problems, small-world networks achieve higher performance and are more robust to random failures in cells than Barabási-Albert networks, though both networks have similar resilience to link removal in networks with many connections.

More evidence about the impact of topology on a system’s dynamics is given by Macêdo *et al.* [53] and Oikonomou and Cluzel [54]. Heverton Macêdo, Gina Oliveira and Carlos Ribeiro [53] evaluated the effect of adding random shortcuts to a network, going from lattices to small-world networks, noticing that more shortcuts lead to more rapid deviation between two cellular automata with similar initial states. Panos Oikonomou and Philippe Cluzel [54] used Boolean threshold networks—a framework similar to cellular automata, but in which connections have weights that can be either positive (excitatory) or negative (inhibitory)—to study how topology may affect the natural evolution of networked systems. Their results indicate that the evolution of systems structured according to scale-free networks is faster and more continuous than that of random networks, which is characterized by large plateaus with sparse improvements in evolution.

A broad analysis performed by Carsten Marr and Marc-Thorsten Hütt [55] used different classes of networks—e.g., rings with different *k*’s and small-world networks—to study how topology can change the behavior of a dynamic system. Their results indicate that, simply by changing the network used to structure interactions between agents, one can make, for instance, an initially homogeneous system to behave chaotically or a system with complex dynamics to enter a periodic regime. In their work, the authors also found that the lower the degree of a node, the more uncertain its behavior—as they have few influences, the aggregated information of these individuals is subject to larger fluctuations. This heterogeneity of behaviors among individuals following the same set of rules is a very interesting finding that we will further explore in this paper.

## Methods

In this work we intended to further investigate how networks defining the interactions in complex systems impact the computation occurring in such systems and their performance when trying to solve problems. In a previous study [17], we have already showed that networks with small-world and scale-free characteristics could enhance the ability of multi-agent systems in collective problem solving. Now we are interested, more precisely, in understanding how information processing is affected by the presence of shortcuts in small-world networks and how topology is correlated with individual behaviors and collective performance.

Therefore, we replicated the experiment proposed by Melanie Mitchell and her colleagues [31], evolving simple CAs to achieve good performance in the density classification task, but with a small and crucial change relative to most of the previous works in literature: instead of evolving only rules, we also evolved the networks that define interactions between cells, keeping fixed, however, the number of shortcuts a CA was able to add to its basic ring network. Evolving rules and network together has a profound impact on the observed results because there is interaction between topology and rules when defining a group’s global behavior [17]: while a network may improve the performance of a system using a specific rule, it can worsen the results achieved by another system in which elements have a slightly different individual behavior.

In many other works trying to explain computation in complex systems, researchers were interested in improving the performance of some CAs and in studying their global properties. We, on the other hand, wanted mainly to observe which kind of individual behaviors emerge and the mechanisms by which the evolved systems shape individuals in order to achieve good collective results. We kept the search procedure as simple as possible, and neither adjusted it nor repeated the process extensively to find the best possible CAs. Thus, we did not expect to achieve results similar to those shown in Table 1. Of particular interest to us is whether the shortcuts affect individual computations, creating asymmetries between cells in cellular automata.

To complete this research we had to choose the mechanism to be used to search for CAs with good performance, the encoding used by such mechanism for rules and networks, and the theoretical framework to understand the evolved behaviors. In the following subsections we will present all decisions made and techniques used.

### Searching good CAs

The space we explored to find cellular automata able to achieve good performance in the density classification problem is overwhelming—in a CA with 149 cells each with 7 connections (the configuration used in this work), in which a ratio of only 0.01 of its connections are rewired, there are about 1.6 ∗ 10^70^ different combinations of rules and topologies. Evaluating all possible CA was not an option! Thus, as many other researchers working with cellular automata, we decided to look for rules with good performance using a genetic algorithm. Beyond the difficulty of designing rules for cellular automata that perform specific computations, a good aspect of using automated search procedures in comparison to using predefined rules is to make us able to find non-intuitive—but suitable—rules about which we would not think by ourselves, showing us some alternative ways a complex system could explore the interactions between multiple agents to achieve collective computation.

Genetic algorithms (GA) [56] are optimization methods inspired by biological evolution. A solution is encoded as a string, known as chromosome, and at each epoch many solutions are evaluated. Those chromosomes with best fitness are preferentially selected for reproduction, producing the population for the next epoch. New chromosomes are created by the combination of genes coming from a pair of parents through crossover and, after that, a small number of randomly selected genes of these new solutions are mutated, aiming at introducing diversity in the population. This process is repeated until a termination criterion is reached (e.g., the maximum number of epochs is reached or the search process stopped finding better solutions). In this process, the better the gene the higher its chance of spreading throughout the population, so that when the execution is finished the population, which was randomly initialized, usually contains high-quality solutions for the problem being solved. It is worth noting, however, that being a metaheuristic, no genetic algorithm can guarantee to find the global optimal solution, whenever a finite amount of computational resources is employed.

**Fig 5:**
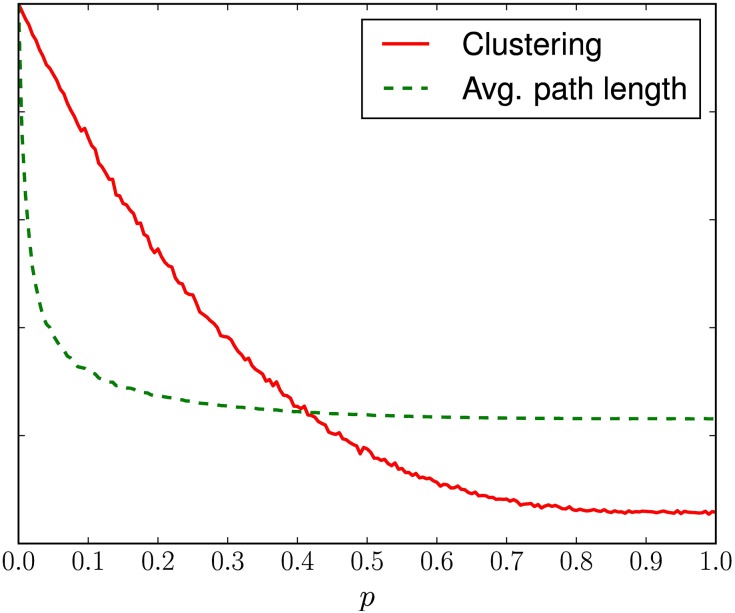
Relative average path length and clustering coefficient in networks generated with the small-world model, according to the rewiring probability *p*.

In our study, the search process was repeated 11 times, each allowing the CAs to have a different number of rewired edges, starting with networks with 0.0 of rewiring, and increasing by 0.01 this ratio at each search, until the ratio of 0.1 was reached. This decision was taken to allow the evaluation of the impact of adding different numbers of shortcuts to the interaction structure of the CA. The maximum value of rewiring ratios was limited to 0.1 because, for a network similar to those used in this experiment, this is the range in which changes in rewiring ratio *p* have the highest impact on topological features, so that small-world characteristics are more prominent, as one can see in Fig 5.

The power of genetic algorithms resides in their ability to explore characteristics common to the structure of many problems, thus we need a proper way to encode solutions into a chromosome for an effective search. Some desirable features of such encoding are: the ability to represent virtually any possible solution of interest to the problem at hand; solutions generated through crossover and mutation must satisfy any existing constraint (e.g.: the in-degree of every cell must be equal to *k*); and solutions with representations close to each other have similar fitness (i.e., the fitness function is smooth). As we needed to represent two different aspects of the cellular automata, we opted for a chromosome composed of two disjoint parts, one representing the topology and the other representing the rule table used.

#### Rule table encoding and rule crossover

In an ECA, each possible set of states a cell and its neighbors may take can be encoded as arrays of zeros and ones [57]: if *k* = 3 and a cell is, currently, in state 0, while its left and right neighbors are, respectively, in states 1 and 0, this input set can be represented by the array 100, or the number 4 if we read this array as a binary number. This means we can list every possible input a cell can see and associate with each of them a unique number. In the case of *k* = 3 we would have 2^*k*^ = 8 possible inputs, numbered between 0 and 7. An even nicer possibility for ECAs is that if we list all output states of a given rule table, sorted by the number associated with its input set, we would have again a binary array and, thus, we could represent each possible rule table a cell can have by a unique number—a representation widely used in literature regarding elementary cellular automata. For instance, if we consider white cells as ones and black cells as zeros, the rule set adopted in Fig 2 would be represented by the array 10001111, and its number would be 143, while the number of the rule in Fig 3 would be 110 (array 01101110).

With the use of irregular networks comes the possibility of cells in a CA having different number of inputs. It would make necessary a different way to encode a rule table. Some researchers [46, 51, 58] solved this problem by adopting a simple rule in which a cell goes to state 1 if a ratio larger than a threshold *τ* of its neighbors are in state 1 and goes to 0 otherwise. However, to keep local characteristics of cells—like rules and number of inputs—as similar as possible, we forced every cell to have exactly *k* = 7 inputs, implying the existence of 2^2^7^^ ≈ 3.4 ∗ 10^38^ possible rule tables for CAs like those we explored in this work. This uniformity imposed that most of the behavioral differences comes from factors external to the cell (externalities) and also allowed us to borrow the same encoding used for ordinary ECAs, making it possible for us to explore more rules than a threshold-based rule would allow.

When generating a new individual, for each entry in the rule table we randomly selected one of the two parents from which we copied the associated output. After that, mutation was performed by flipping each bit with a small probability *π*.

#### Network encoding and topology crossover

As explained, we decided to explore small-world networks [39], but using rewires selected by an evolutionary method, instead of random rewires. In each CA we allowed a fixed number of edges to be reconnected, what means that a CA originally based on a ring network could replace some cells’ original contacts for contacts located anywhere in the system, creating, thus, some shortcuts. Compared to previous analysis, which usually evolved only the amount of rewiring in the network—a parameter not evolvable in our experiments—but not which edge to reconnect, this approach has the advantage of making possible shortcuts to be more carefully placed in the network as well as the evolution of motifs and other control structures.

As a cell can be a source of information for a variable number of peers but can read the state from exactly *k* = 7 neighbors, all networks studied here were directed and edges could only be disconnected from their origins, not from their destinations.

In a CA’s chromosome, the network was encoded by representing only its rewirings. For a given ratio *p* of rewirings, a network is represented by a list with ⌊*p* ∗ *n* ∗ *k*⌋ triples, one indicating each reconnected edge. The first position of such triples stores the index *i* ∈ [1, *n*] of the destination cell of this edge. The second position contains the index *j* ∈ [−*r*, −1] ∪ [1, *r*] of the input edge of the destination cell to be rewired. Here the *j*-th edge is the one that originally pointed from cell *i* + *j* to cell *i*. Edge *j* = 0 represents a self-loop and is not allowed to be rewired, so a cell always considers its own current state when calculating the next state. Finally, the third position indicates the cell *l* ∈ [1, *n*] which is the edge’s new origin. It is relevant to notice that an edge could be rewired back to its previous origin, so that this encoding also allows the evolution to “cancel” rewirings, if it is found to be beneficial to CAs. We did not take any special measure to avoid the occurrence of different representations for isomorphic graphs, considering the computational costs involved in such operation.

Given a pair of parents, a new chromosome was generated by combining the lists of triples from the parents and selecting ⌊*p* ∗ *n* ∗ *k*⌋ unique elements from it. Rewires that occurred in both the parents were twice as likely to be selected for the new individual. Mutation was performed by replacing with probability *π* each triple in the rewire list by a new one randomly selected among all possible rewires. No edge was allowed to be rewired twice. For a given set of parameters *p*, *n* and *k*, these evolutionary operators always generate individuals with the same number of distinct rewirings, so that all solutions produced by crossover and mutation are feasible.

#### Fitness evaluation

Ideally, an individual’s fitness would be given by the proportion of all possible configurations such cellular automaton is able to correctly solve. This is a value extremely difficult to calculate, though, considering that the CAs we evolved have around 7.1 ∗ 10^44^ different possible initial configurations and that, as far as we know, there is no closed method to perform such calculation directly from a CA’s encoding. To estimate an automaton fitness, following Lizier *et al.* [59] we tested it over *c* = 4480 randomly sampled initial configurations. Only tests in which all cells converged to the correct state were considered successful—i.e., a configuration in which more than 0.5 of the cells were initially in state 1 was considered as a failure even if only one cell was in state 0 at the end of the execution. Also following the common practice in literature [31, 34, 37], each CA was executed for *m* = 2 ∗ *n* + 1 = 299 iterations and an execution was considered to converge only if all cells are in the same state during the last and the second-to-last iterations.

The most straightforward way to sample the initial configurations is by defining independently the state of each cell with uniform probability, which yields each possible initial configuration with the same probability. However, as this process gives rise to a binomial distribution of densities with probability *p* = 0.5 of cell being in state 1, most of the configurations generated using this model have densities near *ρ*_0_ = 0.5, the hardest region for the DCT, as changes in initial states of few cells can invert the desired output. To provide an easier landscape to optimize, we used a different sampling mechanism to calculate a CA’s fitness for the evolutionary algorithm: we used a different probability, for each repetition, of initializing a cell with state 1. These probabilities were uniformly spaced in the range (0, 1), as suggested by Melanie Mitchell *et al.* [31].

#### Selection

At each epoch a complete new population was produced. For each of such new chromosomes, its parents were selected based on two different tournaments between 5 random individuals from the previous epoch. The candidate with highest fitness was considered the tournament’s winner and, thus, selected for reproduction.

Table 2 summarizes all configurations used in the experiment.

**Table 2:** Parameters and configurations used in the experiment.

**Genetic algorithm**	
Population	100
Number of epochs	50
Selection method	Tournament
Tournament size	5
Crossover type	Uniform
Mutation rate (*π*)	2%
Rewiring ratios	{0.0, 0.01, …, 0.09, 0.1}
**Cellular automata**	
Size (*n*)	149
Radius (*r*)	3 (*k* = 2 ∗ *r* + 1 = 7 neighbors)
Boundary	Periodic (ring)
Update	Synchronous
Iterations	2 ∗ *n* + 1 = 299
# of repetitions	4480

All simulations were performed using Python 2.7 with NumPy 1.8.2, SciPy 0.13.3 (for statistical calculations) and CUDA 6.5 (for GPU parallel computing) in Ubuntu Linux 14.04 running in an Intel Core i7 CPU (3.50GHz) and a GPU NVIDIA GeForce GTX 660 Ti. The source code for this experiments is available at https://github.com/unicamp-lbic/small_world_ca.

### Understanding collective computation

**Fig 6:**
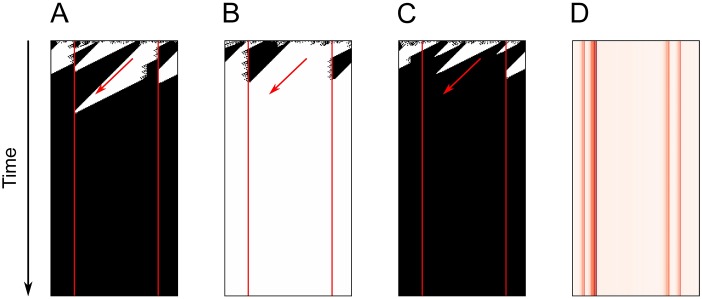
Space-time diagram of executions of the best cellular automaton found with *p* = 0.01 rewired connections, for three different initial configurations. In **(A)**, **(B)** and **(C)**, limits and flow directions are indicated, respectively, by the vertical lines and diagonal arrows. In **(D)**, darker colors indicate that a cell acted more frequently as a limit.

After the examination of space-time diagrams depicting the execution of cellular automata evolved in an initial, exploratory step, we noticed that, unlike reported in works using fixed networks, computation in the evolved CAs was characterized by the occurrence of directed flows and limits—information flows almost freely in a specific direction until it encounters a limit, a cell that “decides” whether the flow will continue or will be interrupted. In the evolved CAs, the cells which act as limits are very stable over many different initial configurations (see Fig 6).

To understand such different behaviors, in the second phase of our work we evolved new CAs following the same setup used before, but evaluating topological conditions—as the occurrence of rewiring, degrees, clustering coefficient, distances and centrality measures—for the emergence of such limits and whether they were related to higher performance. To determine whether a cell acted as a limit in a CA, we estimated the direction and velocity that better approximate the flow of information in the CA and, then, counted in all repetitions how frequently a cell interrupted the flow of information, acting as a limit. For each CA, the estimated flow was given by the integer *f* that minimized the difference between the automaton states in iteration *t* rotated by *f* ∈ [−*r*, *r*] positions and the automaton states in iteration *t* + 1. Given *f*, a cell *i* is considered to interrupt the flow of information in a given iteration if its state differs from the state of cell *i* − *f* in the previous iteration. Fig 6 shows the limit measure calculated for a CA with ratio 0.01 of rewiring.

**Fig 7:**
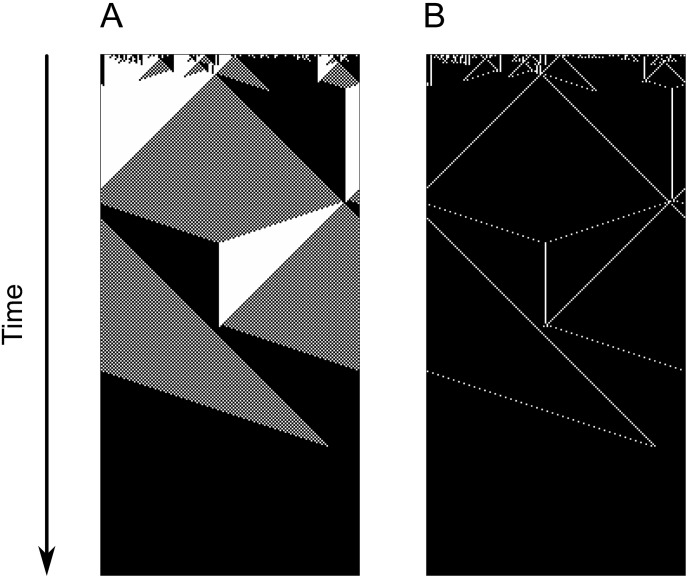
Space-time diagram for an execution of the GKL rule [35] (with regular ring topology) and particles observed during such execution.

Previous works with uniform and regular ECAs have established different analytic frameworks to explain how computation happens in cellular automata. *Computational mechanics* [33, 49, 60] is an analogy with particle physics which explains computation in CAs by using particles as information carriers and collisions between particles as computational events. As the reader can see in Fig 7, particles are defined by boundaries between two different kinds of domains and they travel through the CA transporting information about local densities and sizes of high- and low-density regions. When particles collide, they can both annihilate each other or emit new particles, depending on the types of the colliding particles. However, despite being helpful to predict the behavior of a CA from its rule table and understand its errors [61], this approach is not suited to explain the behavior of irregular networks as the notion of space, central to computation mechanics, is destroyed in small-world networks with the addition of connections between cells not adjacent in the original ring.

Manuel Marques-Pita and Luis Rocha [62] proposed a second way to analyze cellular automata, known as *schema redescription*. The main idea is to eliminate redundancy in rule tables for cellular automata, leaving only input states that are relevant to define a cell’s next state and grouping states in which the exact position of an input is irrelevant (the only information that matters in this group is the number of cells in state 0 or in state 1). After this redescription, the rule table keeps only the input states that effectively determine the automaton’s transitions, which reduces the number of rules to analyze, making it easier to detect symmetries in a CA’s behavior. Using this technique, Marques-Pita and Rocha were able to show that, despite showing different global behaviors, the hand-designed rule GKL [35] is extremely similar at local level to the rule GP, found by Andre, Bennet and Koza [34] using genetic programming.

Considering its ability to evaluate cells based on their observed behaviors and not only on their rule tables, our framework of choice is a set of information theoretic measures proposed by Joseph Lizier, Mikhail Prokopenko and Albert Zomaya [63, 64] to analyze local dynamics of information in distributed systems like cellular automata and random Boolean networks. They presented a set of measures intended to indicate how information is stored, transferred and modified in the system—operations required for universal computation.

The *stored information* that is currently used by a cell to calculate its next state is given by its *active information storage*. It is defined as the average mutual information between the semi-infinite past of cell *i* and its next state at time step *t* + 1:

$$\begin{array}{c} A_{i}=\lim_{j\to \infty }\left\langle \log_{2}\frac{p(x_{i,t}^{\left(j\right)},x_{i,t+1})}{p\left(x_{i,t}^{\left(j\right)}\right)p\left(x_{i,t+1}\right)}\right\rangle , \end{array}$$ (1)

in which *x*_*i*,*t*_ is the state of the cell *i* at time *t* and $x_{i,t}^{\left(j\right)}$ is the *j* past states of cell *i*, from time *t* − *j* + 1 to time *t*. Considering that it is not feasible to compute *A*_*i*_ in the limit *j* → ∞, the approximation *A*_*i*_(*j*) with finite history length *j* is used. *A*_*i*_ can be either positive or negative indicating, respectively, if the cell’s current state is relatively likely or unlikely, considering its past. The reader can also notice that *A*_*i*_ can assume high values even if the cell *i* does not have a connection to itself through a self-loop, as it can store information in a distributed manner, using its neighbors.

Different measures have been proposed to evaluate different aspects of the *information transfer* to a cell from its neighbors. We decide to use the *local collective transfer entropy*, which captures the amount of information transferred from all causal sources that were not already contained in the cell’s past. In deterministic systems, like CAs, its value is equal to the *local entropy rate* [64], which can be calculated as:

$$\begin{array}{c} H_{\mu i}=\lim_{j\to \infty }H_{x_{i,t+1}|x_{i,t}^{\left(j\right)}}, \end{array}$$ (2)

in which $H_{x_{i,t+1}|x_{i,t}^{\left(j\right)}}$ is cell *i*’s *conditional entropy*, the uncertainty about the cell next state when its *j* previous states are known:

$$\begin{array}{c} H_{x_{i,t+1}|x_{i,t}^{\left(j\right)}}=-\sum_{x_{i,t+1},x_{i,t}^{\left(j\right)}}p\left(x_{i,t+1},x_{i,t}^{\left(j\right)}\right)\log_{2}p(x_{i,t+1}|x_{i,t}^{\left(j\right)}). \end{array}$$ (3)

Again, as the computation of the limit for *j* → ∞ is not feasible, a finite *j* is used to calculate the local collective transfer entropy.

The local entropy for a cell is the sum of the local active information and the local entropy rate:

$$\begin{array}{c} H_{i}=A_{i}+H_{\mu i}, \end{array}$$ (4)

a relation preserved even for approximations with finite *j*.

To measure *information modification*, the framework proposes a metric named *local separable information* which, however, can be considered more a heuristic, as it double-counts parts of the information in the next state of the destination. A proper metric is still under development [65] and, therefore, local separable information is not used in our work.

Lizier and colleagues applied their framework to study information flow in cellular automata and random Boolean networks [63, 65], quantitatively verifying, for instance, the conjectures about roles of particles and collisions in the computational mechanics framework [63]. They also evaluated information dynamics in random Boolean networks [59], discovering that, while regular and random topologies are usually dominated, respectively, by information storage and information transfer, RBNs based on small-world networks balance both storage and transfer capacity.

## Results

**Fig 8:**
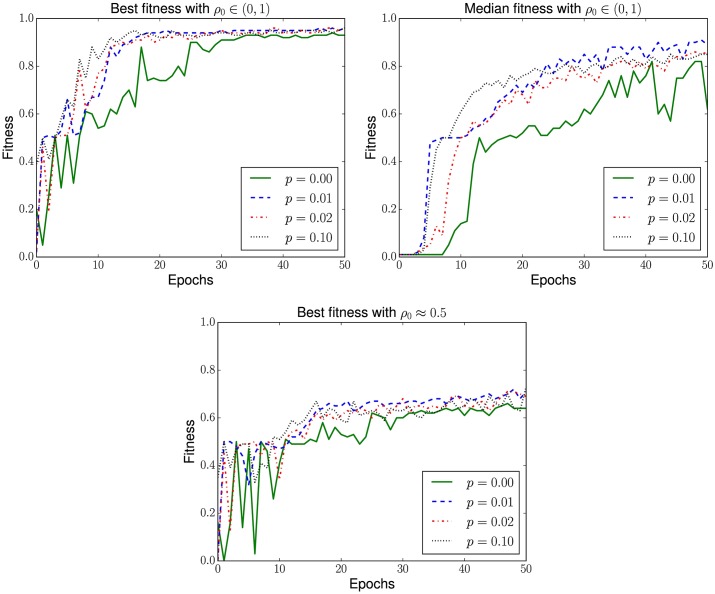
Evolution of fitness during the searches with different rewiring probabilities *p*. The plots indicate, for each epoch, the best and the median fitness of the population, calculated with initial configurations with densities evenly distributed in the range *ρ*_0_ ∈ (0, 1). We also show the results achieved by the best individuals in each epoch when initial configurations are sampled with density *ρ*_0_ ≈ 0.5.

In Fig 8 we show, for each different rewiring probability *p*, the evolution of the distribution of fitness in the population during the search. The first thing the reader can notice is that, similarly to results seen in the literature [47], the evolution was faster in finding multiple good solutions when we allowed the topology to be rewired: in searches with *p* > 0 we observed a general improvement of fitness around the 4th epoch, while for fixed topologies such improvement was seen only after 8 epochs. It is worth noticing that all searches were successful in finding rules able to solve more than 90% of the initial configurations, which were sampled with densities evenly distributed in the range *ρ*_0_ ∈ (0, 1). We also show in Fig 8 the performance of the best individual at each epoch, testing them in initial configurations sampled with density *ρ*_0_ ≈ 0.5, the most common and, also, most difficult situation in the DCT. Again, all the best results achieved when allowing rewiring—respectively, 72%, 71%, 71%, 69%, 71%, 67%, 74%, 72%, 71% and 73%—were better than the obtained with fixed topology—when 66% of all initializations were correctly solved.

**Fig 9:**
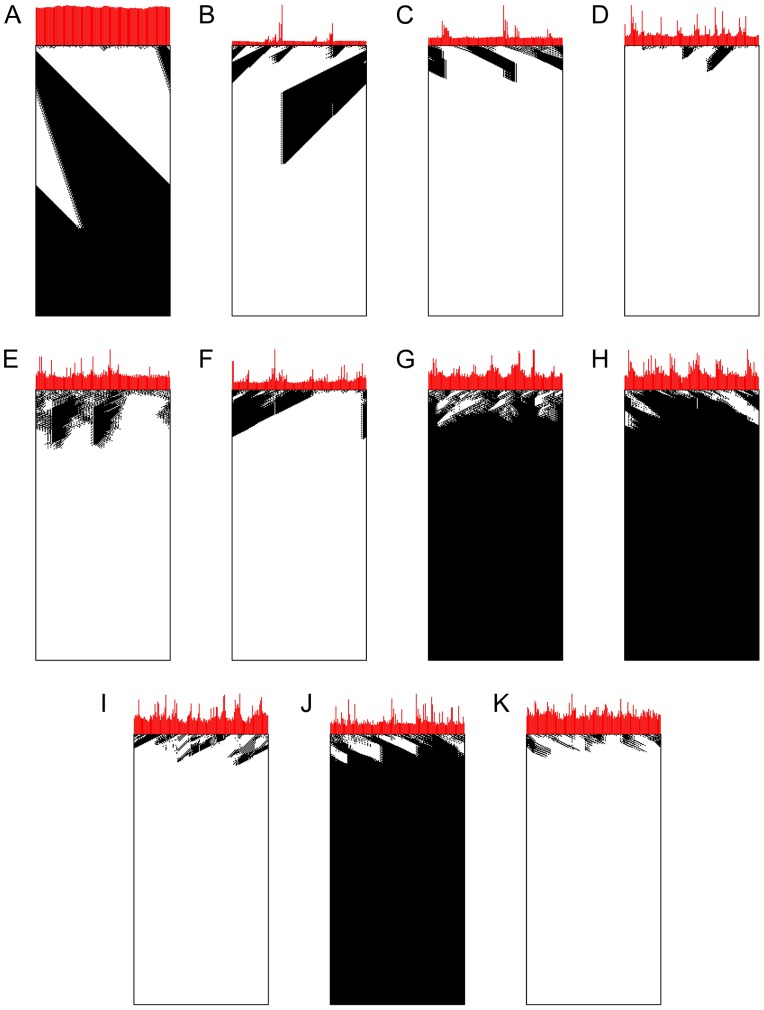
Space-time diagram of executions of the best cellular automaton found at each execution. Above each space-time diagram is depicted a bar graph indicating how often each cell acted as a limit. To improve visualization the bar graphs were scaled so the maximum height is equal across all CAs, so that bar heights should be compared solely within each graph. **(A)** *p* = 0.00, **(B)** *p* = 0.01, **(C)** *p* = 0.02, **(D)** *p* = 0.03, **(E)** *p* = 0.04, **(F)** *p* = 0.05, **(G)** *p* = 0.06, **(H)** *p* = 0.07, **(I)** *p* = 0.08, **(J)** *p* = 0.09 and **(K)** *p* = 0.10.

We exhibit in Fig 9 space-time diagrams with examples of executions of the best individuals found for each different *p*. When *p* = 0 the observed behavior is very similar to that of particles from computational mechanics, though we did not achieve rules so refined as those obtained by Melanie Mitchell and colleagues [31], with many different domains and particles. We remember, however, that in their work, from hundreds of executions, only few searches were able to find such rules with high performance. As more edges are allowed to be rewired, we can observe the loss of spatial coherence.

**Fig 10:**
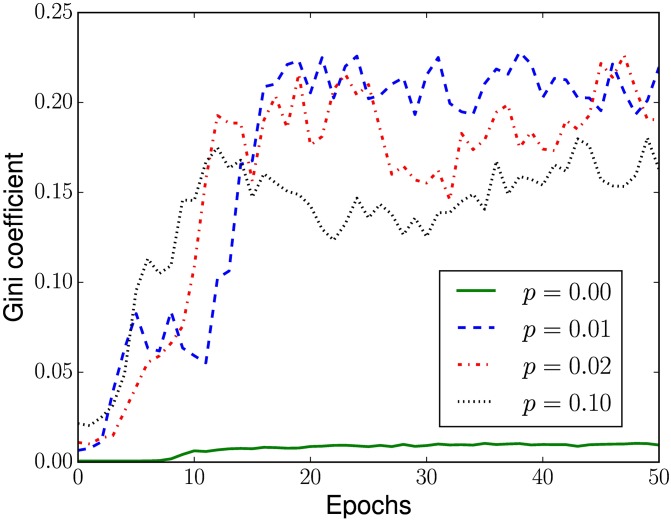
Evolution of the Gini coefficient. Evolution of median inequality of frequencies that cells in each CA acted as limits during the search with different rewiring probabilities *p*.

**Fig 11:**
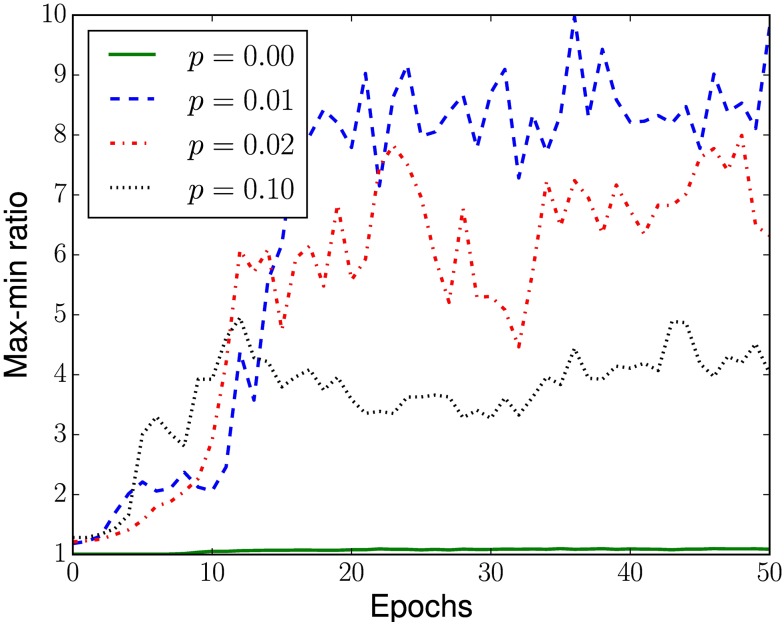
Evolution of the ratio between the maximum and minimum frequencies a cell acted as a limit. Evolution of median ratio between the maximum and minimum frequencies that cells in each CA acted as limits during the search with different rewiring probabilities *p*.

At the top of each space-time diagram, we also plotted a bar graph indicating how often a cell acted as a limit. Even though such measure is only a heuristic, in these graphs it is possible to see that, in each CA, there are groups with few cells that act as limit at least twice as often than the others, a fact even more prominent for smaller *p*. This is evidenced in Fig 10, which displays the evolution of inequality in the role performed by the cells during evolution, measured with the Gini coefficient [66]. Gini coefficient is a summary statistic that measure the inequality in a population of non-negative reals, assuming values between zero (when all individuals are equal) and one (when only one individual has value different from 0). We can notice that, consistently across all executions with *p* > 0, as the population’s fitness increases fewer cells will act as limits (apparently) “deciding” the fate of information flows. At the final epochs of the evolutionary search, such most decisive cells act, usually, 4 to 10 times more often as limits than the least decisive ones (see Fig 11).

### Inequality, topology and fitness in cellular automata

In Table 3, we can see how some general metrics regarding the cellular automata’s efficacy and structure correlate with the inequality in behavior among cells. In addition to clustering coefficient and average path length, we also indicate inequality in out-degrees (the number of edges originated in each node) and in closeness centrality (a measure that indicates how close a given node is to all other nodes in the network). The first point to notice is the high correlation between inequality and a CA’s fitness, agreeing with what was seen in Figs 8 and 10. For every search, such correlation was stronger than that between fitness and average path length (max|*ρ*| = 0.302), which may indicate that, in the evolved CAs, the presence of limits was more relevant for a successful automaton than a small average distance between cells. Also, in almost every execution, there was statistically significant indication of a weak negative correlation between inequality in the behavior of the cells and both average clustering coefficient and average path length. Such results indicate that CAs where the occurrence of limits was concentrated in fewer cells also favored rewires in which the new source of information was not in the neighborhood of any other source of the destination cell (i.e., the rewired connection brings new information to such cell). Regarding inequality in out-degrees, also measured using the Gini coefficient, in 7 of 10 executions we can see a weak negative correlation with concentration of limits in fewer cells. This may indicate that inequality in the behavior of the cells is associated with a lower presence of hubs that broadcast information to many cells; however, more research is needed to confirm such hypothesis. We highlight that most of the correlations discussed before were not seen in the execution for *p* = 0.01. We conjecture that the evolutionary process took a different and (apparently) less likely path during this search. It is important to have in mind that most of the observed correlations are weak and that the experimental design does not allow us to look for causal relations between the metrics, so that we cannot confirm or rule out whether these associations are mediated only by the correlation to a third variable (fitness, for instance).

**Table 3:** Spearman’s rank correlation between the inequality of limits distribution and CA’s metrics. Statistically significant correlations (*p*_*value*_ < 10^−5^) are marked with ‘*’.

Rewire (*p*)	Fitness	Clustering	Avg. Path Length	Out-degree (Gini)	Closeness (Gini)
0.00	0.609*	–	–	–	–
0.01	0.512*	0.157*	0.064*	0.015	0.130*
0.02	0.555*	−0.073*	−0.143*	0.058	−0.034
0.03	0.537*	−0.079*	−0.066*	−0.144*	−0.042
0.04	0.531*	−0.106*	−0.072*	0.071*	−0.075*
0.05	0.549*	−0.134*	−0.173*	−0.162*	−0.049
0.06	0.414*	−0.035	0.006	−0.007	0.026
0.07	0.539*	−0.172*	−0.155*	−0.083*	−0.014
0.08	0.569*	−0.158*	−0.110*	−0.217*	0.027
0.09	0.554*	−0.060	−0.047	−0.118*	0.064*
0.10	0.419*	−0.017	−0.008	−0.008	0.014

The results for *p* = 0 need a more careful explanation: in such situation, despite all cells having the same pattern of connections and following the same rule table—which makes inequality in behavior virtually impossible—we see a strong correlation between behavior inequality in a CA and its fitness. This is an artifact of the heuristic used to detect limits in the space-time diagram, as it also identifies as limits many domain boundaries in the computational mechanics paradigm. However, as the reader can see in Fig 10, the Gini coefficient when *p* = 0 is much smaller than the values observed when *p* > 0. Indeed, this correlation is a spurious result from the finite number *c* of repetitions used to estimate fitness and behaviors and it fades away as *c* → ∞.

### Cells’ roles and their position within the network

Now, it is important to understand whether such limits, in fact, play a relevant computational role in the evolved cellular automata. For this, we used the information theoretical metrics presented in the previous section and evaluated their Spearman’s rank correlation with the value indicated by the limit heuristic. For each different CA, the information theoretical metrics were calculated for each cell based on their behaviors in all of the 4480 different initial configurations tested. We observed a strong correlation between the frequency a cell acts as a limit and its local entropy rate (*ρ* = 0.691, *p*_*value*_ ≪ 10^−10^), while the limit frequency is negatively correlated with the active information storage (*ρ* = −0.473, *p* ≪ 10^−10^). Such results agree with our initial hypothesis that cells that work more as limits have a different role when compared to the other cells. The high correlation with local entropy indicates that the limits are important for the integration of information from different places, while the other cells function more as the cellular automaton’s memory.

Let us then investigate possible reasons for this difference in behavior between cells. The first thing we will do is to evaluate the Spearman’s rank correlation between the value indicated by our limit heuristic and structural metrics about the underlying topology of the CA (for *p* > 0). For this, we used basic local metrics—a cell’s in- and out-degrees, its local clustering coefficient, the average out-degree of its neighbors and the number of rewired input edges—and also classical centrality measures [67, 68], that are affected by a cell’s position in relation to the whole network. The centrality measures used are:

- PageRank, HITS and eigenvalue centrality, which, in our design, measure how much information can flow to each node. PageRank and eigenvalue centrality are computed using the recursive assumption that the information a node can gather is proportional to the information gathered by this node’s neighbors. HITS (Hypertext Induced Topic Search), in turn, provides two different values for each node (“authority” and “hub” scores) indicating respectively how influenced and how influential is a node. The basic (recursive) idea in HITS is that nodes with more “authority” are pointed by good “hubs” and that good “hubs” point to nodes with more “authority”.
- Closeness centrality considering both the original and the reversed network, to evaluate respectively how fast information flows from and to the node.
- Load and betweenness, that indicate the fraction of shortest paths in the network that pass through each node, measuring how central a node is for efficient connections in the network.
- Eccentricity, i.e., the node’s maximum distance to all other nodes.

Despite all correlations achieving statistical significance (*p*_*value*_ ≪ 10^−10^), we did not observe any strong correlation between the measures and the frequency a cell acts as a limit. However, we saw a weak correlation (|*ρ*| ≈ 0.1) of limits with the number of rewired inputs (*ρ* = 0.141) and with a node’s closeness centrality in the reversed network (*ρ* = 0.130). We also observed a negative correlation with clustering coefficient (*ρ* = −0.113), which is expected as the rewiring process tends to reduce local clustering coefficient.

**Fig 12:**
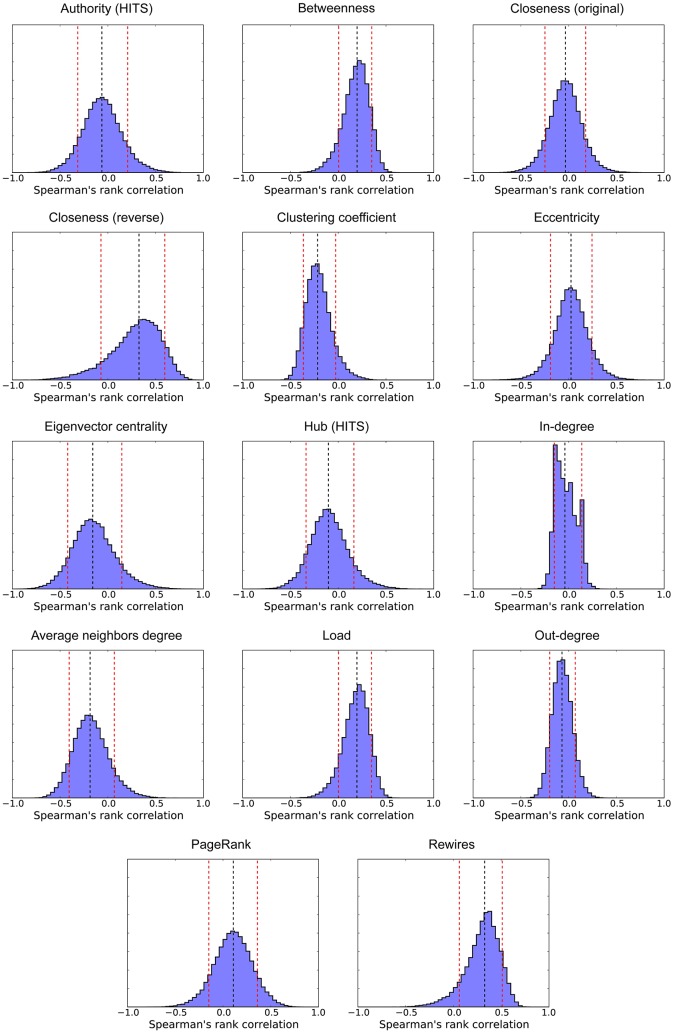
Distribution of spearman’s rank correlation, calculated for each CA, between the frequency a cell acts as a limit and the cell’s structural metrics. The black vertical line indicates median value and the red vertical lines indicate, respectively, the 10th and the 90th percentiles (*N* = 51000).

We also looked at this relationship between a cell’s behavior and its position within the CA’s topology from a different perspective: instead of considering all cells in all CAs to calculate the correlations between the frequency a cell is a limit and its structural metrics, we computed such correlations for each CA separately (for *p* > 0). Our goal is to better understand the factors that explain why, in each specific CA, some cells act regularly as limits and others do not. Fig 12 shows histograms depicting the distribution of correlations we observed. As we can see, the number of rewired inputs and the closeness centrality in the reverse graph have a moderate median correlation with limits ($\tilde{\rho }=0.327$ and $\tilde{\rho }=0.326$, respectively), agreeing with our previous observations. As closeness in the reverse graph and number of rewires are strongly correlated ($\tilde{\rho }=0.530$), we evaluated Spearman’s partial rank correlation between frequency as limit and closeness in the reverse graph, controlling for rewired inputs, obtaining a relevant residual effect ($\tilde{\rho }=0.187$). Weaker correlations are also observed with betweenness ($\tilde{\rho }=0.197$) and load ($\tilde{\rho }=0.196$) centralities. Such results suggests that cells that act more frequently as limits are usually those able to gather information from different regions of the automaton more quickly, being relevant not only the number of rewired inputs a cell has, but also the regions accessed by the cell through such links. As a side effect of the rewiring process, negative correlations are observed for clustering coefficient ($\tilde{\rho }=-0.216$) and average neighbors out-degree ($\tilde{\rho }=-0.186$).

### How edges are reconnected

After looking at the cellular automata from macroscopic (focusing CAs’ general metrics) and microscopic perspectives (analyzing cells’ characteristics), we will assume an intermediary point of view, analyzing local patterns of connections between cells (motifs) associated with CAs with high fitness. Using the ESU algorithm [69], which enumerates all subgraphs with size *k*, we counted how many times each pattern of connections with *k* = 3 and *k* = 4 cells occurred in the best CA found in each execution with *p* > 0. Subgraphs in the same isomorphism classes were grouped. We used only one graph per search to avoid counts to be artificially inflated by the dependency between solutions in an execution of an evolutionary search, as these methods work by replicating blocks found in the previous epoch, usually (but not exclusively) associated with good fitness. Therefore, even a harmful motif, found in the initial population or generated by mutation, could be present in many CAs of a progeny (and, thus, identified as a motif) before the evolutionary pressure eliminated it.

**Fig 13:**
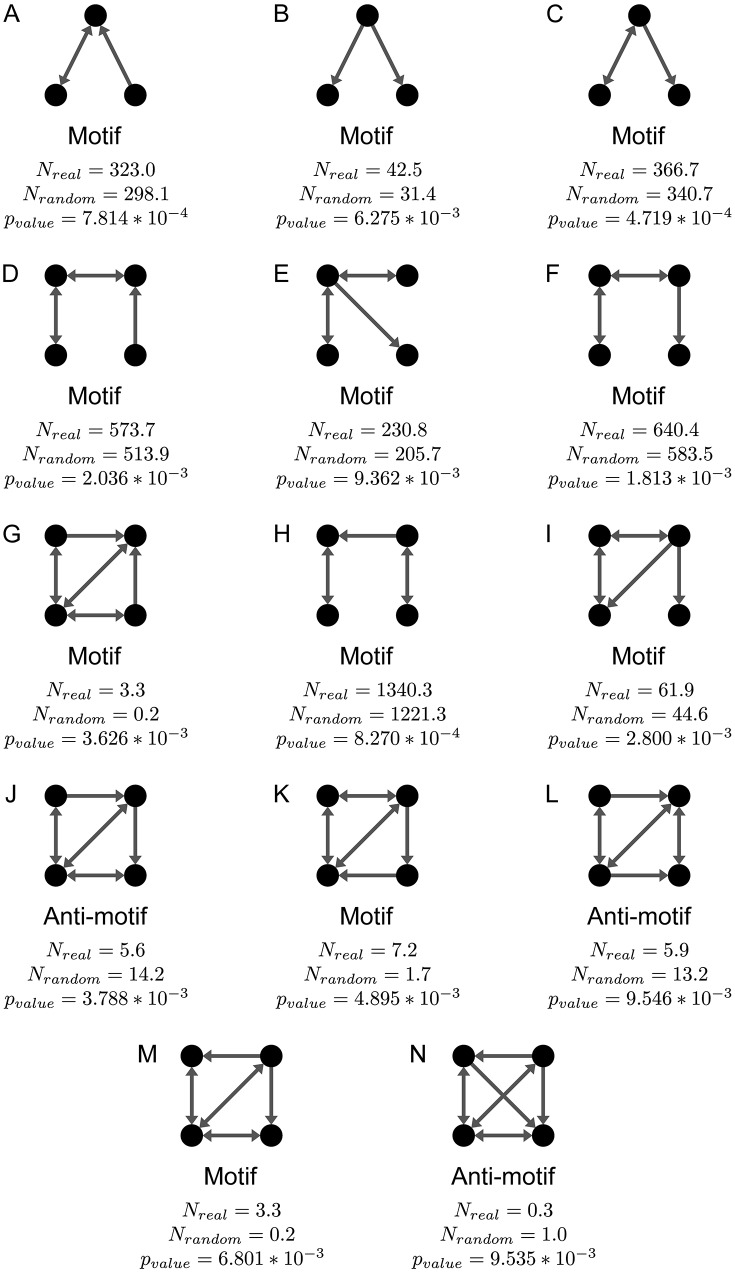
Motifs identified in the best topologies found in each search. *N*_*real*_ is the average count of the respective subgraph in each network. *N*_*random*_ is the average number of occurrences of such subgraph in similar networks, but with random rewires.

For each selected graph, we generated an exact copy, but with random rewires, which we used as a baseline to check whether a subgraph is a motif or an *anti-motif* (i.e., it appears significantly less than the expected). Such evaluation was made using a paired t-test for each subgraph, comparing the number of occurrences in the original graph and in the baseline. In Fig 13 we exhibit all the subgraphs of size 3 or 4 whose number of occurrences in the high fitness graphs was significantly different from the occurrences in the randomized graphs (*p*_*value*_ < 10^−2^). We call attention to the fact that we lowered the bar for statistical significance in this analysis considering the low number of graphs analyzed (only 10 for each motif). Even though we raised the significance threshold, we point that the probability of false positives producing results similar to those reported in this work, with at least 3 false positives in 13 comparisons (for motifs of size 3) and 11 false positives in 199 comparisons (for motifs of size 4) with *p*_*value*_ ≤ 10^−2^, is of only 2.653 ∗ 10^−4^ and 6.563 ∗ 10^−6^, respectively (assuming independent comparisons).

From the motifs depicted in Fig 13, we can infer the occurrence of four different general patterns of reconnections in cellular automata with high fitness:

- Long range rewires: motifs A, C, D, E, F and H indicate that rewires creating edges between nodes that were not previously in the same neighborhood are more likely in cellular automata with higher fitness.
- Multiple rewires per cell: motif B can be created either by the addition of edges from the central node or the removal of edges directed to it. Considering the absence of any motif of size 4 corroborating to the additive hypothesis and also motifs I and M, the most likely process is the rewiring of at least two of the central node edges originally coming from nodes that had no direct connection between each other.
- Centralization of rewires in a neighborhood: the observed anti-motifs J, L and N share one feature—all of them are likely to be generated by at least two different nodes that were directly connected in the original topology rewiring some of their input edges. Such situation may be the result of benefits obtained by a CA when it centralizes in only one node per neighborhood the task of integrating information arriving from different places of the CA.
- There and back again: motif K is likely to be created by the addition of a size 2 path by which the information coming from a neighborhood flows to a distant node, where it is integrated with information from other parts of the CA, and then it flows back to its original neighborhood.

We highlight that all the patterns observed in this motif analysis are in line with the correlations pointed in the previous subsections, as both analyses point to the importance of rewires to bring information from other regions of the CA and to the concentration of multiple rewires towards few cells, which integrate information from different places.

## Discussion

We observed in the experiments performed during this study that, unlike previous works that used traditional elementary cellular automata with ring topology, when making the topology more flexible different classes of cells emerge with qualitatively different computational behaviors. In traditional ECAs that solve the density classification task, the movement of information structures—particles and domains—and their collisions are the main mechanisms for information transmission and modification, so that any type of computational process can happen anywhere in the CA. On the other hand, in cellular automata that allow edges to be rewired we saw that, usually, information flows in specific directions, and decisions about interrupting or allowing the propagation of such flows are made by specific cells, which we named limits. It is remarkable that even systems with individuals as simple as the cells in ECAs can achieve labor division—and obtain the benefits that come with it—only by creating and removing few connections between cells.

Differently from most works in emergent computation [62], we focused not on general characteristics of the systems under analysis, but on understanding how local interactions can introduce specialization and, thus, produce the desired collective computation. After analyzing the behavior of limits according to information theoretical measures, we saw that they have the role of integrating information from multiple regions of the cellular automaton while other cells are, mainly, devoted to store information. This is a very interesting result from the point of view of network science, considering that all cells follow the exact same rules and have the exact same number of inputs, so any difference in behavior that is persistent over multiple initializations of a CA can only be attributed to differences among the positions these cells occupy in the CA’s underlying structure. The observed specialization is also curious, as the evolutionary algorithm found, with no guidance, a method to overcome the lack of separation between memory and processing units of cellular automata, partially mimicking the separation between storage and data processing seen in systems as von Neumann computers.

Our analyses were not conclusive about which network characteristics contribute for the emergence of specialization in a cellular automaton. We were able, however, to gather coherent evidence pointing to some patterns and properties that are likely to have an important role in defining whether a CA will show limits and, also, which cells will act as limits. Considering the negative correlation of inequality with average distance between cells and clustering coefficient and, also, the patterns of connections in motifs found in CAs with high fitness, the presence of long-distance rewires seems to be a relevant factor for the occurrence of stronger limits. Limits are usually cells that have the ability to quickly gather signals coming from different regions—what is reflected in a positive correlation of limits with closeness centrality in the reverse graph—and, thus, tend to have many of their input edges rewired to distant cells and to centralize all rewires in their neighborhoods.

These results are in tune with those reported by Lizier and colleagues [59] about computational capabilities of Boolean networks based on small-world, regular and random topologies. They observed a strong correlation between networks with high clustering coefficient and information storage and a negative correlation between information transfer and average distances in a network, which points to the importance of long links for information transfer in a decentralized system. Small-world networks, according to their analysis, display a balance between information storage and transfer, which may render them suitable for complex computation. Our results indicate that these computational capabilities are not uniformly distributed across the network, but rely on network-induced specialization. Combining this capacity of interaction networks to affect global computation and individual behaviors with other results from network science [39, 48, 70, 71], we can wonder whether networks structuring many complex systems have some similar properties not only due to coincidence or to physical restrictions, but that they were positively selected by evolution or learning to do (at least) part of the computation performed collectively. There is already evidence of this evolutionary design of social networks in humans, for instance, as researchers have observed direct influence of some genes on the propensity of two individuals developing a long-term relationship [72].

While in neuroscience the idea of heterogeneity emerging from differences in connections is not new, they bring a new perspective when applied to other complex systems, particularly to social networks (both involving humans or other animals). Considering the variety and intricacy of individual behaviors, when analyzing social systems scientists usually think that complexity can prescind network structure or regard it to a secondary role in relation to individuals. However, as the difference of position in a network can partially explain the existence of individual diversity even in systems as simple as cellular automata, a proper understanding of collective behaviors may require consideration of the structure of interactions between agents, even when evidence indicates that individual behaviors are the sole responsible for a computation. This focus on social relations echoes many ideas from distributed cognition [9, 73], which argues that mental processes are not restricted to single human brains but extend also to interactions with other people and the environment.

Finally, as the correlations we observed between behavior and network properties are either weak or moderate, a deeper study is necessary in the future in order to investigate other requisites for the emergence of inequality and specialization in elementary cellular automata with non-uniform topology. Despite not being able to generate limits when there is no heterogeneity in topology, a CA’s rule table is also crucial for the existence of different classes of cells—as a trivial example, a rule in which a cell only repeats its previous state would never allow the emergence of limits, no matter the underlying network used. So, in future works it is relevant to better explore whether different types of specialization may emerge when different rules are applied and how sensitive are cells’ computational behaviors to changes in the rules they follow. In order to advance the study reported here towards more complex systems, particularly social networks, it is also important to investigate how collective computation and the emergence of diverse behaviors is impacted by richer underlying networks—e.g.: networks that include more than one type of interaction, allow the exchange of complex signals, and support relations with different intensities.
